# Supplementary material for: Functional Characterization of PeVLN4 Involved in Regulating Pollen Tube Growth from Passion Fruit
Source: Int J Mol Sci. 2025 Mar 6;26(5):2348. doi: 10.3390/ijms26052348 (PMC11899883; doi:10.3390/ijms26052348)
Supplement: Supplementary file 1 [file ijms-26-02348-s001.zip › ijms-3489736-supplementary/Supplemental figures with legends.pdf]

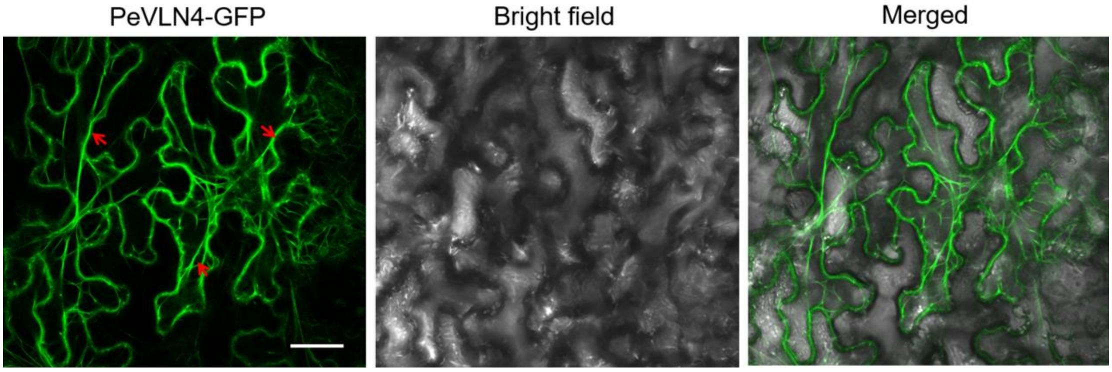

Supplemental figure 1. Subcellular localization of PeVLN4 in *N. benthamiana*. The construct of *pCambia1301-35Spro-PeVLN4-GFP* was transiently expressed in *N. benthamiana*. Bar=50 μm. Red arrows indicate filamentous structures.

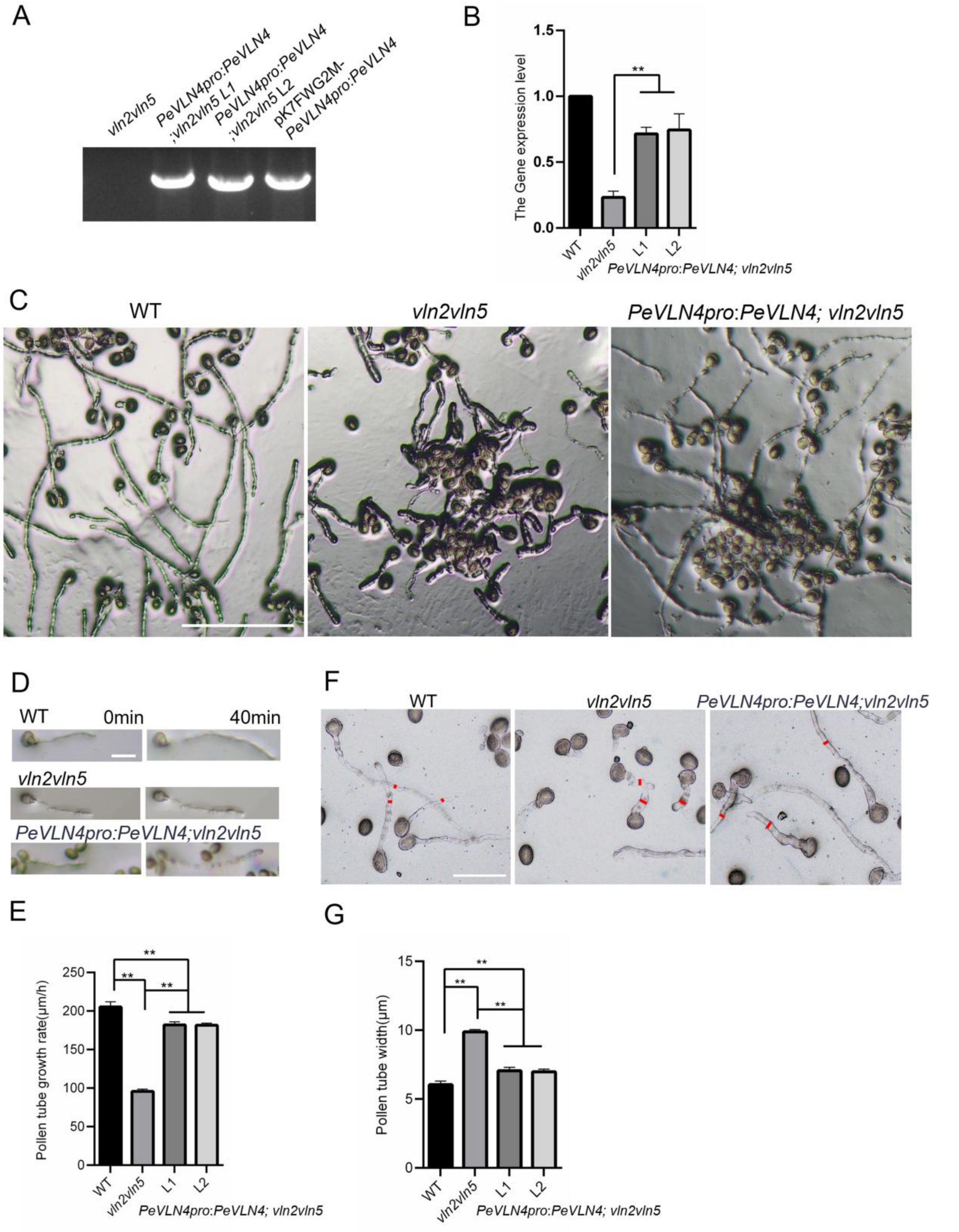

Supplemental figure 2. PeVLN4 is crucial for pollen tube growth and shape.(A) PCR verification for wild-type Col-0, *vln2vln5* mutant, transgenic lines expressing *PeVLN4* and the plasmid as a positive control. Lanes 1, 2, 3, and 4 represent *vln2vln5*, transgenic line 1, 2 and the plasmid.(B) Quantitative real-time PCR analysis for Col-0, *vln2vln5* mutant, transgenic lines expressing *PeVLN4*. The expression level of the *eIF4A* gene was used as an internal control. Data are presented as mean ± SD (n = 3); **P < 0.01 (Student's *t*-test).(C) Micrographs of pollen tubes after germination for 3 h. Pollen was isolated from plants with the following genotypes: wild-type Col-0 ,*vln2vln5* mutant, and homozygous transgenic line. Bar =100 μm. (D) Micrographs of the pollen tube at two different time points. Bar= 50 μm.(E) Quantification of the growth rate of the pollen tubes. Data are presented as mean ± SD (n = 3).

Pollen tube growth rate was calculated for Col-0 ,*vln2vln5* mutant, and homozygous transgenic lines. ** P < 0.01 (Student's *t*-test).(F) Micrographs of the pollen tube shape from each genotype. Bar= 50 μm. Red lines indicate pollen tube width. (G) Quantification of the width of the pollen tubes. Data are presented as mean ± SD (n = 3). Pollen tube width was calculated for Col-0 ,*vln2vln5* mutant, and homozygous transgenic lines. ** P < 0.01 (Student's *t*-test).
