# Supplementary material for: Functional Characterization of PeVLN4 Involved in Regulating Pollen Tube Growth from Passion Fruit
Source: Int J Mol Sci. 2025 Mar 6;26(5):2348. doi: 10.3390/ijms26052348 (PMC11899883; doi:10.3390/ijms26052348)
Supplement: Supplementary file 1 [file ijms-26-02348-s001.zip › ijms-3489736-supplementary/Table S1 List of primers used in the article.pdf]

Supplemental Table 1

List of primers used in the article

Name	Primer sequence(5'-3')	Purpose
qPeV4F	CCCGGTCTGTCAAAGCAAGC	qRT-PCR analysis to detect *PeVLN4* transcripts
qPeV4R	CTCTCGCTTGGTCACGTCAA	
qPeV4-1F	GTCCTGGACTGTCACTCTGA	qRT-PCR analysis to detect *PeVLN4-1* transcripts
qPeV4-1R	CCCAGGAAAAAAATCGAGTG	
qPeV4-2F	CTTGTGAGTGAAGCAAGCCC	qRT-PCR analysis to detect *PeVLN4-2* transcripts
qPeV4-2R	CAGTGACAGGATCTGATGAGTT	
EF1AF	TCCCTGACAAAGCAGAAGATG	qRT-PCR analysis to detect *EF1A* transcripts as an internal control in *Arabidopsis* pollen
EF1AR	GATGTAGCAGCCTACCTGAAAG	
PeV4F	ATGGCTGTTTCCATGAGAGA	PCR verifying *PeVLN4* in transgenic lines
PeV4R	GAACAACTGAAGGGCCATTT	
EcoRI -PeV4-F	AGCAAATGGGTCGGGATCCGAATTC GATGTTTTTCTTCACTGGATTG	Cloning *PeVLN4* CDS to generate protein expression construction of *pET23b-PeVLN4*
SalI- PeV4-R	TCGAGTGCGGCCGCAAGCTTGTCG ACGAACAACTGAAGGGCCATTT	
PeV4pro-Flap	AGAAGCTTGCATGCCTGCAGGTCG ACGAGTAACTCACGGGGGTCTG	Overlapping cloning *PeVLN4* promoter and CDS to generate *pK7FWG2M-PeVLN4pro:PeVLN4-GFP*
PeV4pro-Rlap	TCTCTCATGGAAACAGCCATATTGT ATAGAAGCACCAACTAT	
PeV4CDS-Flap	AGTTGGTGCTTCTATACAATATGGCT GTTTCCATGAGAGA	
PeV4CDS-Rlap (PKME)	CCTCGCCCTTGCTCACCATTGATATC TTAGAACAACTGAAGGGCCA	
PeV4CDS-Rlap (PKM)	CCTCGCCCTTGCTCACCATTGATATC TTGAACAACTGAAGGGCCATTT	PeV4pro-Flap/PeV4pro-Rlap and PeV4CDS-Flap/PeV4CDS-Rlap(PKM) were used to ovelapping cloning *PeVLN4* promoter and CDS sequence to generate *pK7FWG2M-PeVLN4pro:PeVLN4*
qPev4pollenF	TTTCCATGAGAGATTTGGAT	qRT-PCR analysis to detect *PeVLN4, PeVLN4-1, PeVLN4-2* transcripts in passion fruit pollen
qPev4pollenR	ATAGGAATCCCCTGTGAAAA	
eIF4AF	GGGTATCTATGCTTACGGTTTCG	qRT-PCR analysis to detect *eIF4A* transcripts as an internal control in *Arabidopsis* pollen
eIF4AR	CAGAGAACACTCCAACCTGAATC	
XbaI-35spro-PeV4-F	CTATCTCTCTGCAGGTCGACTCTAG AATGGCTGTTTCCATGAGAGATTT	Cloning *PeVLN4* CDS to generate *pCambia1301-35Spro:PeVLN4-GFP*
SacI-35spro-PeV4-R	CAAATGTTTGAACGGAATTCGAGCT CTTACTTGTACAGCTCGTCCATGC	
