# Supplementary figures and images for: Functional Characterization of PeVLN4 Involved in Regulating Pollen Tube Growth from Passion Fruit
Source: Int J Mol Sci. 2025 Mar 6;26(5):2348. doi: 10.3390/ijms26052348 (PMC11899883; doi:10.3390/ijms26052348)

PeVLN4-GFP

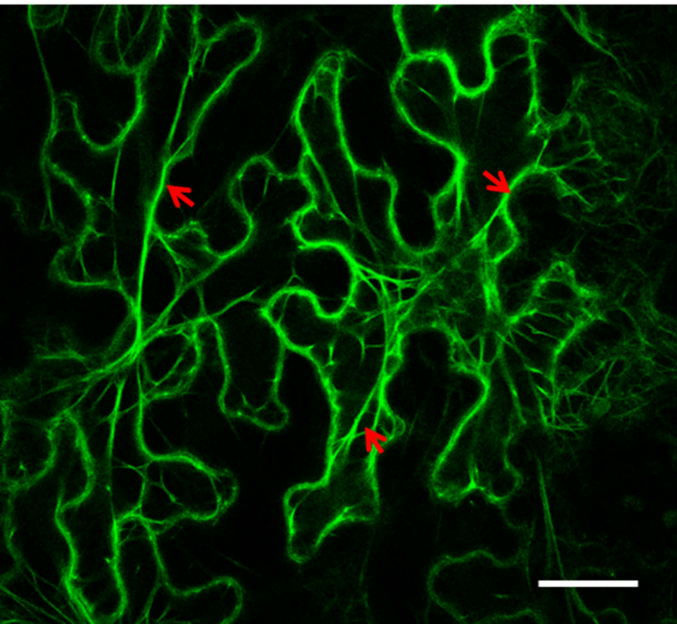

Bright field

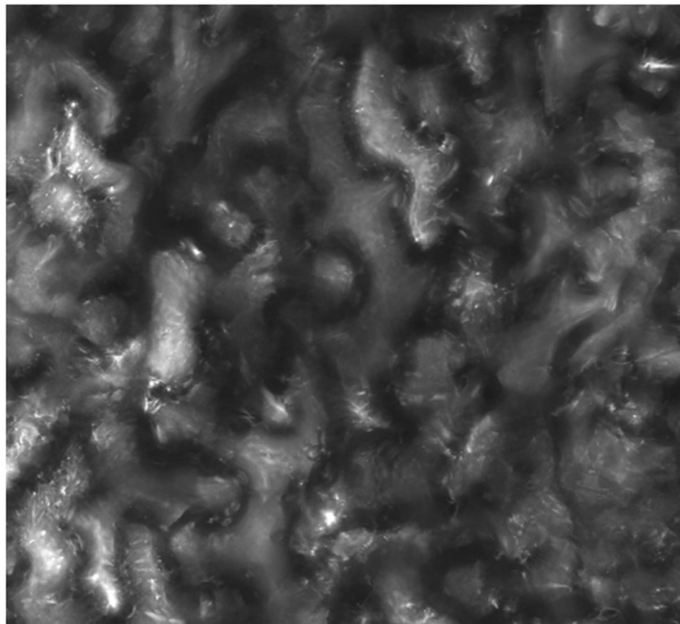

Merged

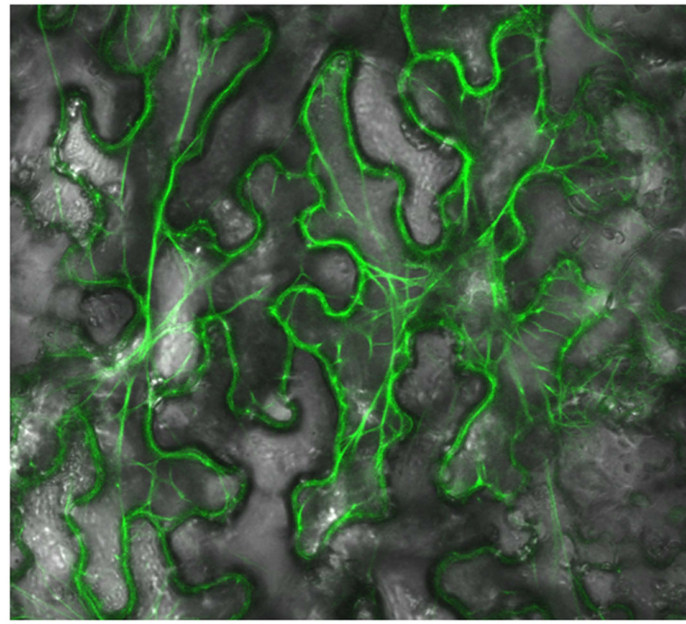

Supplement: Supplementary file 1 [file ijms-26-02348-s001.zip › ijms-3489736-supplementary/Figure S1 Subcellular localization of PeVLN4 in N. benthamiana.pdf]

A

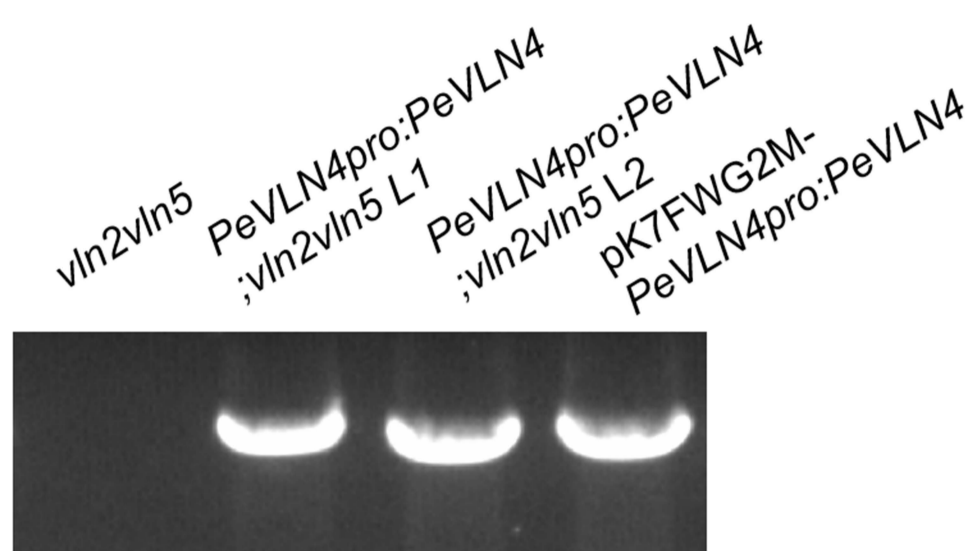

B

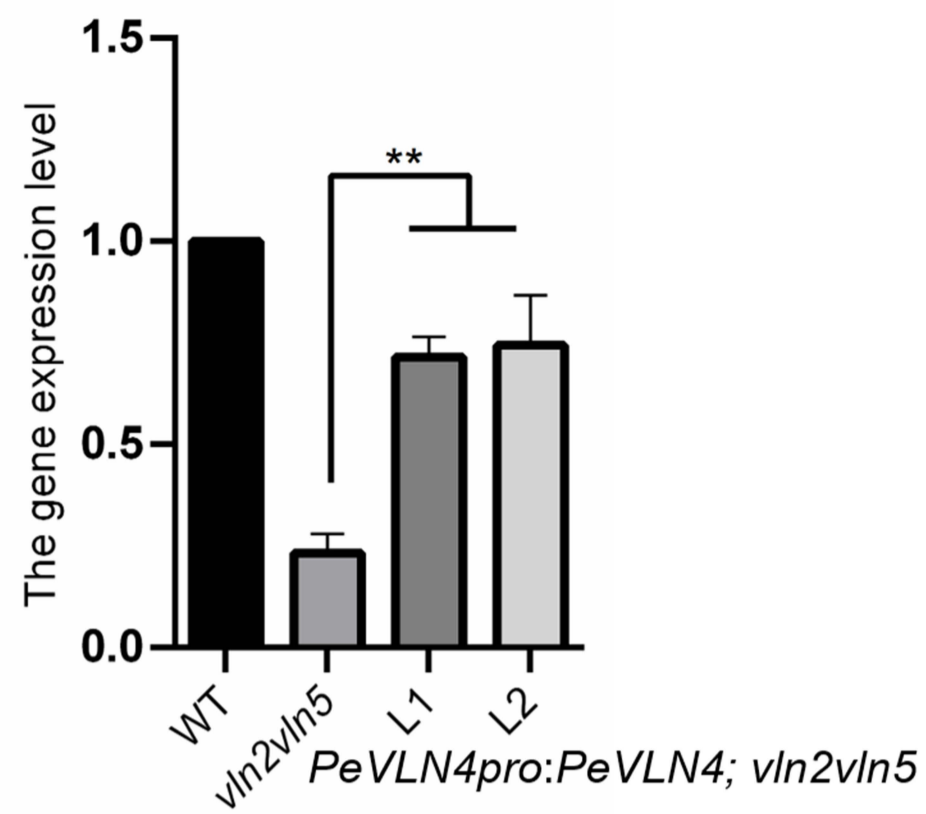

C

WT

*vln2vln5**PeVLN4pro:PeVLN4; vln2vln5*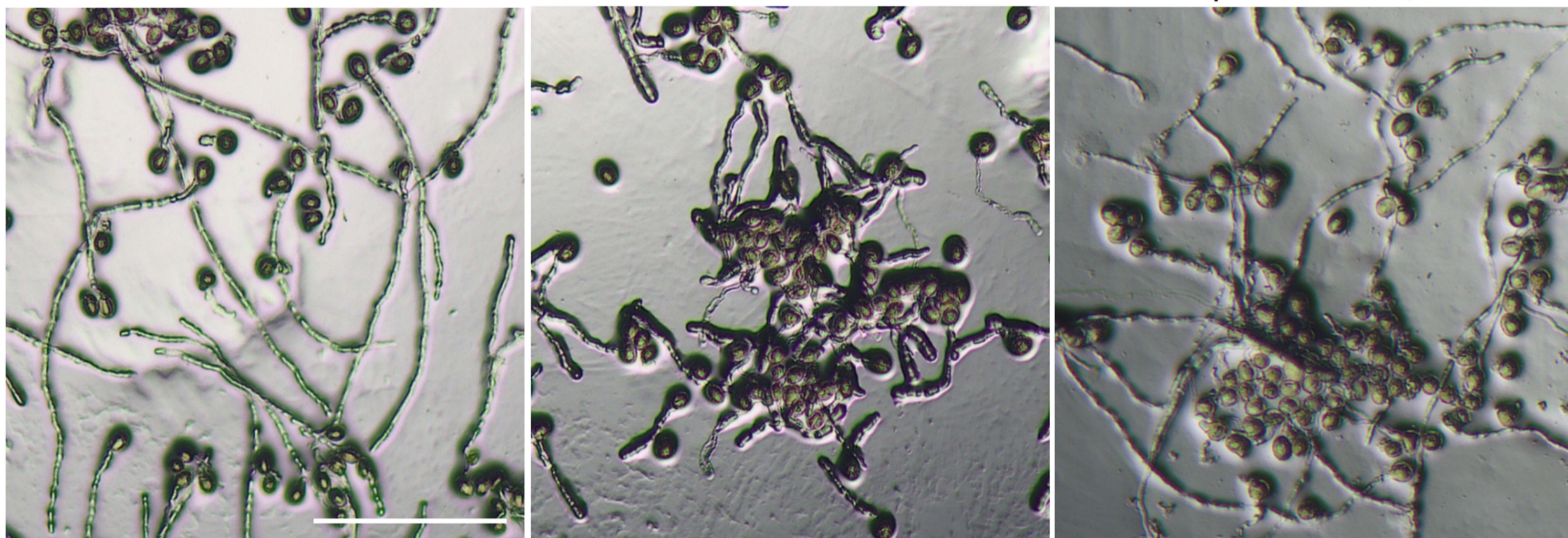

D

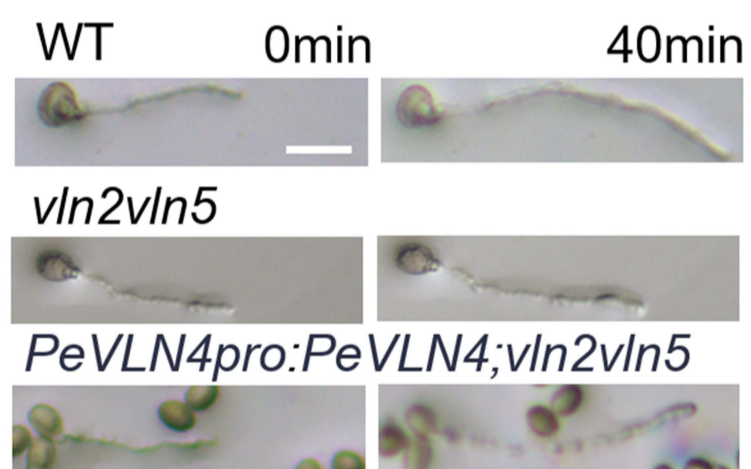

F

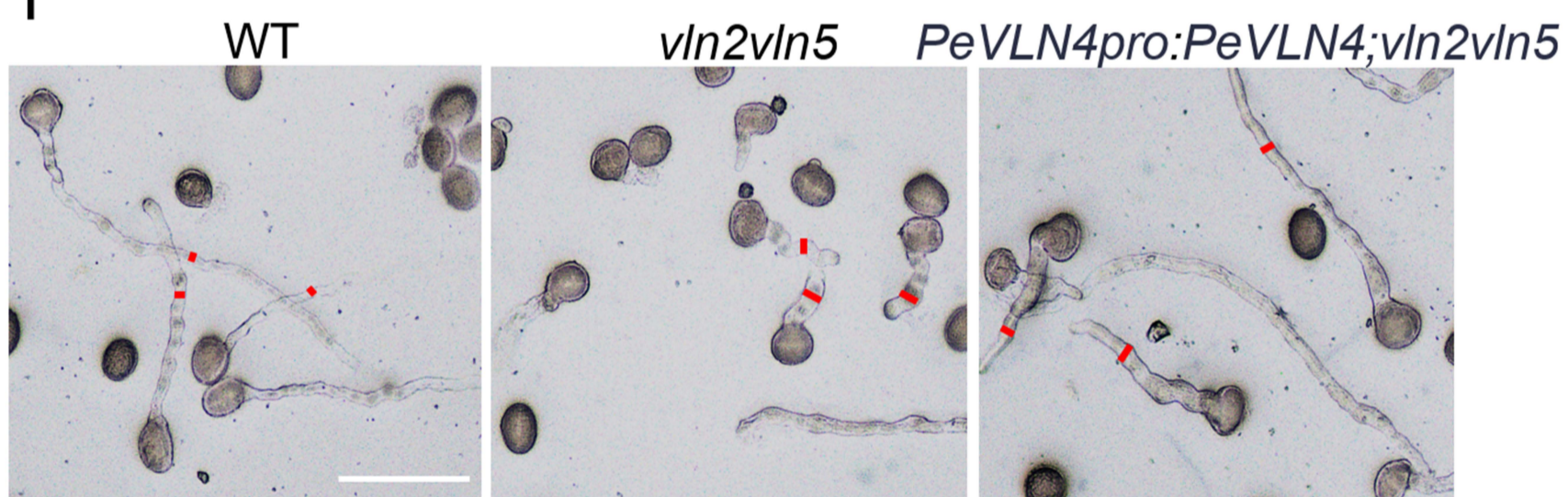

E

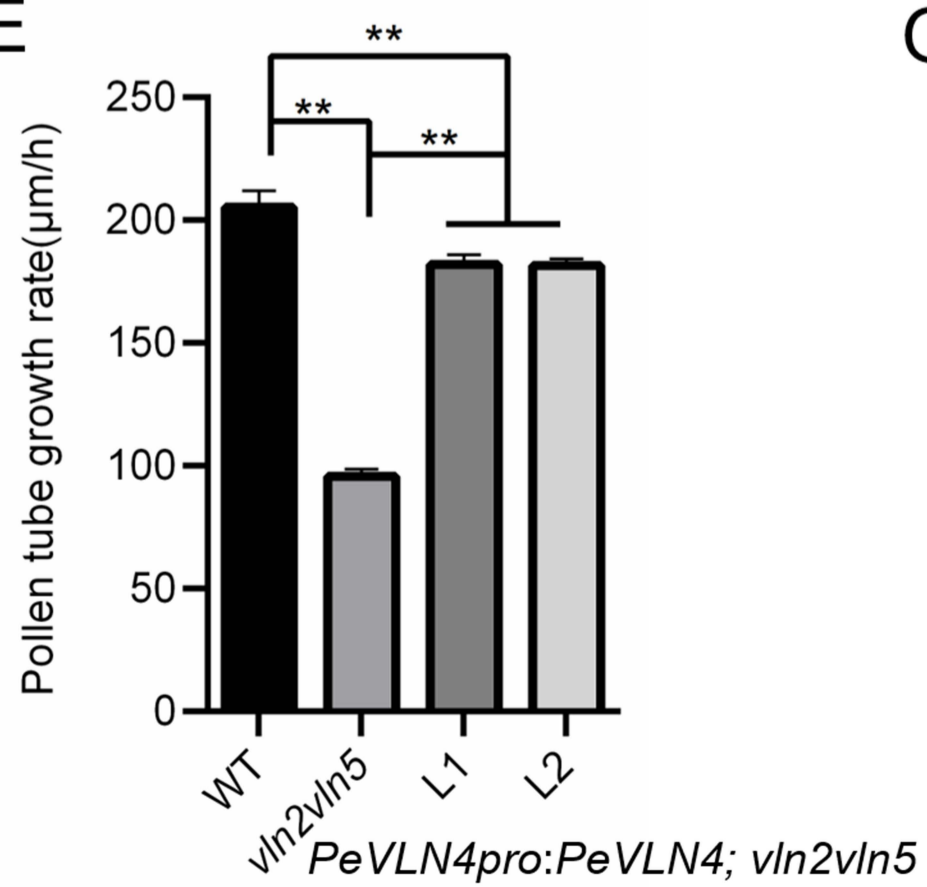

G

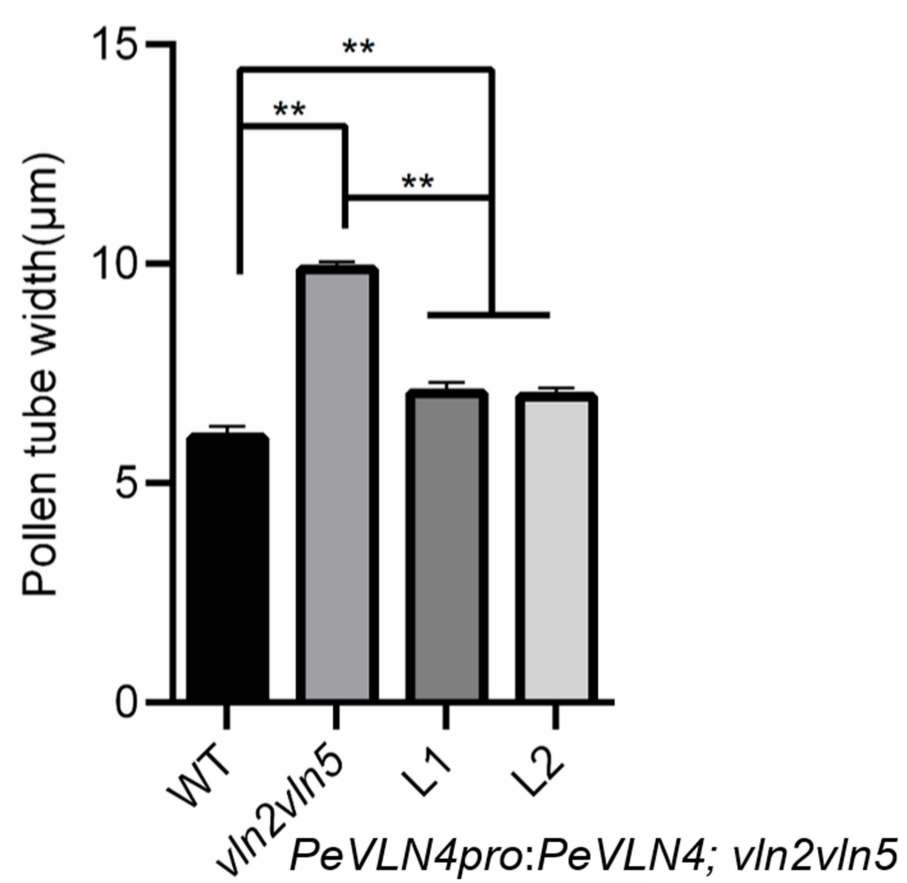

Supplement: Supplementary file 1 [file ijms-26-02348-s001.zip › ijms-3489736-supplementary/Figure S2 PeVLN4 is crucial for pollen tube growth and shape.pdf]
